# Development and Evaluation of Quantitative Immunoglobulin G Enzyme-Linked Immunosorbent Assay for the Diagnosis of Coronavirus Disease 2019 Using Truncated Recombinant Nucleocapsid Protein as Assay Antigen

**DOI:** 10.3390/ijerph18189630

**Published:** 2021-09-13

**Authors:** Pierre Nsele Mutantu, Mya Myat Ngwe Tun, Takeshi Nabeshima, Fuxun Yu, Patrick Kakoni Mukadi, Takeshi Tanaka, Masato Tashiro, Ayumi Fujita, Nobuhiro Kanie, Ryosaku Oshiro, Takahiro Takazono, Yoshifumi Imamura, Tatsuro Hirayama, Meng Ling Moi, Shingo Inoue, Koichi Izumikawa, Jiro Yasuda, Kouichi Morita

**Affiliations:** 1 Graduate School of Biomedical Sciences, Nagasaki University, 1-12-4 Sakamoto, Nagasaki 852-8523, Japan; pierremutantu@gmail.com (P.N.M.); patrickmukadi@gmail.com (P.K.M.); 2 Department of Virology, Institute of Tropical Medicine, Nagasaki University, 1-12-4 Sakamoto, Nagasaki 852-8523, Japan; myamyat@tm.nagasaki-u.ac.jp (M.M.N.T.); mtmikami@tm.nagasaki-u.ac.jp (T.N.); sherry@nagasaki-u.ac.jp (M.L.M.); moritak@nagasaki-u.ac.jp (K.M.); 3 Program for Nurturing Global Leaders in Tropical and Emerging Communicable Diseases, Nagasaki University, 1-12-4 Sakamoto, Nagasaki 852-8523, Japan; 4 Guizhou Provincial People’s Hospital, Guiyang 550002, China; yufuxun@126.com; 5 Department of Clinical Medicine, Institute of Tropical Medicine, Nagasaki University, 1-12-4 Sakamoto, Nagasaki 852-8523, Japan; 6 Infection Control and Education Center, Nagasaki University Hospital, 1-7-1 Sakamoto, Nagasaki 852-8501, Japan; ttakeshi@nagasaki-u.ac.jp (T.T.); mtashiro@nagasaki-u.ac.jp (M.T.); ayumi.f@nagasaki-u.ac.jp (A.F.); koizumik@nagasaki-u.ac.jp (K.I.); 7 Department of Infectious Diseases, Nagasaki University Hospital, 1-7-1 Sakamoto, Nagasaki 852-8501, Japan; nobubububble@gmail.com (N.K.); o.ryosaku@gmail.com (R.O.); 8 Department of Respiratory Medicine, Nagasaki University Hospital, 1-7-1 Sakamoto, Nagasaki 852-8501, Japan; takahiro-takazono@nagasaki-u.ac.jp (T.T.); yimamura@nagasaki-u.ac.jp (Y.I.); tatsuro_h_20@nagasaki-u.ac.jp (T.H.); 9 Medical Education Development Center, Nagasaki University Hospital, 1-7-1 Sakamoto, Nagasaki 852-8501, Japan; 10 Department of Emerging Infectious Diseases, Institute of Tropical Medicine, Nagasaki University, 1-12-4 Sakamoto, Nagasaki 852-8523, Japan; j-yasuda@nagasaki-u.ac.jp

**Keywords:** SARS-CoV-2, nucleocapsid protein, IgG indirect ELISA, plaque reduction neutralization test

## Abstract

Severe acute respiratory syndrome coronavirus 2 (SARS-CoV-2) is the causative agent of coronavirus disease 2019 (COVID-19). Real-time RT-PCR is the most commonly used method for COVID-19 diagnosis. However, serological assays are urgently needed as complementary tools to RT-PCR. Hachim et al. 2020 and Burbelo et al. 2020 demonstrated that anti-nucleocapsid(N) SARS-CoV-2 antibodies are higher and appear earlier than the spike antibodies. Additionally, cross-reactive antibodies against N protein are more prevalent than those against spike protein. We developed a less cross-reactive immunoglobulin G (IgG) indirect ELISA by using a truncated recombinant SARS-CoV-2 N protein as assay antigen. A highly conserved region of coronaviruses N protein was deleted and the protein was prepared using an *E. coli* protein expression system. A total of 177 samples collected from COVID-19 suspected cases and 155 negative control sera collected during the pre-COVID-19 period were applied to evaluate the assay’s performance, with the plaque reduction neutralization test and the commercial SARS-CoV-2 spike protein IgG ELISA as gold standards. The SARS-CoV-2 N truncated protein-based ELISA showed similar sensitivity (91.1% vs. 91.9%) and specificity (93.8% vs. 93.8%) between the PRNT and spike IgG ELISA, as well as also higher specificity compared to the full-length N protein (93.8% vs. 89.9%). Our ELISA can be used for the diagnosis and surveillance of COVID-19.

## 1. Introduction

Coronavirus disease 2019 (COVID-19) is an emerging viral disease caused by the novel coronavirus severe acute respiratory syndrome coronavirus 2 (SARS-CoV-2). The virus was first reported in Wuhan City, China, in late 2019 and rapidly spread in the country and throughout the world [1]. As of 9 March 2020, COVID-19 cases were reported in more than 100 countries [2]. Subsequently, the disease was declared a pandemic by the World Health Organization (WHO) on 11 March 2020 [3]. At the beginning of the COVID-19 outbreak, molecular techniques were first applied to detect the viral genome using conventional reverse transcription-polymerase chain reaction (RT-PCR) and deep sequencing [4,5,6]. Currently, real-time RT-PCR is considered the most commonly used technique for diagnosing COVID-19 [7]. Nevertheless, molecular methods rely on the timing of sample collection, adequate sample collection technique, and a sufficient amount of viral RNA from the sample collection site [4]. Reliable serological assays are urgently needed as complementary tools to molecular techniques to enhance the capability of laboratory diagnosis [8,9], especially for individuals with viral load undetectable by real-time RT-PCR [10]. Furthermore, the detection of SARS-CoV-2 asymptomatic carriers who have a strong transmission capacity remains critical, especially in resource-limited regions. In such situations, reliable, convenient, and cost-effective diagnosis methods such as serologic tests [11] are highly needed for COVID-19 diagnosis, understanding the immune response to SARS-CoV-2 [12,13], and epidemiological surveillance [14].

SARS-CoV-2 is an enveloped, positive-sense RNA virus with an approximately 30-kilobase genome size that belongs to the *Coronaviridae* family, in the *Betacoronavirus* genus [15]. The SARS-CoV-2 genome contains a minimum of six open reading frames (ORFs) [12]. Like other coronaviruses, the SARS-CoV-2 genome encodes four major structural proteins, including spike (S), envelop (E), membrane (M), and nucleocapsid (N) [1] and sixteen non-structural proteins (nsp 1-16) [12]. Among these proteins, N and S are the main antigens used for serological diagnosis of coronaviruses [16,17,18]. The structural protein N is smaller than S protein and lacks glycosylation sites; it is involved in assembly by binding to the viral RNA genome [2,12]. Although its immunological importance is not well known, this protein plays a key function in regulating the viral RNA transcription during the replication stage [19]. The N protein encounters the host immune system after dissociating from the viral genome inside the cell. However, the S protein specifically binds to the receptor of the host cells through its receptor-binding domain (RBD) both before, during, and after the infection has been initiated [19]. These properties make it a good target antigen for the detection of neutralizing antibodies [12] as well as for enzyme-linked immunosorbent assay (ELISA) [14,20].

Before the COVID-19 outbreak, six members of the *Coronaviridae* family were known to infect humans. Two of these species, severe acute respiratory coronavirus (SARS-CoV) and Middle East respiratory syndrome coronavirus (MERS-CoV), cause severe respiratory illness, whereas four are responsible for common cold symptoms (human coronavirus [HCoV] HKU1, OC43, NL 63, and 229E) [21]. These human coronaviruses (HCoVs) share 90.5%, 46.1%, 27.6%, 26.5%, 20.0%, and 19.1% amino acid homology with SARS-CoV-2 N protein, respectively [4].

Most serological tests have focused on the S protein antibody detection method [13], which may not detect asymptomatic SARS-CoV-2 carriers eight weeks after the infection has occurred [22]. In addition, the performance of S protein might be affected by the mutations resulting from the immune pressure [12]. In contrast, the smaller size of N protein, including the absence of glycosylation sites, makes cloning easier by prokaryotic expression systems [12]. Moreover, antibodies to N protein are generated earlier than S protein [12,23,24]. However, N protein might generate false-positive results [25].

This study aimed to develop a less cross-reactive IgG SARS-CoV-2 N protein-based ELISA, which is faster and cheaper than the current RT-PCR. The cross-reactivity with other human coronaviruses was reduced by deleting the first 121 residues of N protein, which contains a highly conserved motif (FYYLGTGP) of all coronaviruses [26,27]. The reliability of the assay was evaluated using the plaque reduction neutralization test (PRNT) and the commercial S protein-based IgG indirect ELISA.

## 2. Materials and Methods

### 2.1. Human Samples

Over 600 clinical samples were collected from suspected COVID-19 cases in 2020 in Nagasaki, Nagasaki City, Japan, for virological and serological diagnoses. Nasopharyngeal swab samples were used to detect viral genes by real-time RT-PCR [6] and/or reverse transcription loop-mediated isothermal amplification (RT-LAMP) assay [28]. Of these, 149 were laboratory confirmed as either symptomatic or asymptomatic positives cases, and the remainder were diagnosed as negative cases. Among them, EDTA blood samples were collected one month later in 2020 from 177 people for serological diagnosis. In this group, 139 real-time RT-PCR and/or RT-LAMP positive samples were included. In addition, 100 serum samples collected from febrile illness cases and 55 serum samples collected from healthy volunteers in 2007 were used for the pre-COVID-19 group. All 332 plasma/serum samples were heat-treated at 56 °C for 30 min before applying ELISA and PRNT.

### 2.2. Virus Inoculation and RNA Extraction

SARS-CoV-2 strain 2019-nCoV/Japan/TY/WK-521/2020 (not registered in the GenBank) that was isolated in Tokyo from migrants from Wuhan in January–February 2020 [29] was inoculated to confluent monolayer Vero E6 cells maintained at 37 °C in Eagle’s minimum essential medium (MEM) containing 2% fetal calf serum (FCS) and 0.2 mM nonessential amino acids. Infected culture fluid (ICF) was harvested by centrifugation (2000× *g* for 10 min) on day 4 when strong cytopathic effects (CPE) appeared (80% to 100%). Viral RNA was extracted from the ICF by using a QIAamp viral RNA mini kit (Qiagen, Hilden, Germany) according to the manufacturer’s instructions and stored at −80 °C until use.

### 2.3. Construction of SARS-CoV-2 Truncated and Full-Length Nucleocapsid Recombinant Plasmids

SARS-CoV-2 RNA was used as a template to generate the SARS-CoV-2 N truncated gene, in which the first 121 amino acids (aa) were deleted (N_Δ121_) by real-time RT-PCR using Takara PrimeScript one-step RT-PCR kit (Takara Bio, Kusatsu, Japan).

The SARS-CoV-2/human/CHN/HS_194/2020 MT081068.1 strain was used to design the In-fusion primer sets 5′-TCACCATCACGGATCCCTGCCGTACGGTGCTAA-3′ and 5′-TTGGCTGCAGGTCGACTCAAGCCTGGGTAGAGT-3′, which were used to amplify the upstream sense region and the downstream anti-sense region of the SARS-CoV-2 N_Δ121_ gene, respectively. For the SARS-CoV-2 full-length rN gene construct, the forward primer 5′-TCACCATCACGGATCCATGTCTGATAATGGCCCC-3′ with the reverse rN_Δ121_ primer was used. The expected size of the N_Δ121_ and full-length N RT-PCR product was confirmed after electrophoresis on the agarose gel. The PCR product was then purified from the agarose gel using a QIAEX II Gel Extraction Kit (Qiagen GmbH, Hilden, Germany). The pQE-30 plasmid DNA was extracted from the Lauria-Bertani medium (LB broth) containing µg/mL ampicillin *E. coli* culture fluid by using a Qiagen plasmid mini kit (Qiagen GmbH, Hilden, Germany) and digested with BamHI (Takara Bio, Kusatsu, Japan) and SalI (Takara Bio, Kusatsu, Japan) restriction enzymes. Finally, the plasmid backbone was purified from the agarose gel after gel electrophoresis using a QIAEX II Gel Extraction Kit (Qiagen GmbH, Hilden, Germany) and treated with calf intestinal alkaline phosphatase (CIAP) (Takara Bio, Kusatsu, Japan).

The purified SARS-CoV-2 N_Δ121_ or full-length N gene product was then cloned into the pQE-30 plasmid vector using the In-fusion method (5x In-Fusion HD Enzyme Premix, Takara Bio, Kusatsu, Japan) and then transformed into *E. coli* XL-1 Blue. The selection of positive clones containing the SARS-CoV-2 N_Δ121_ or full-length N gene from the *E. coli* colonies that appeared on the LB agar plate was performed by PCR. The nucleotide sequence of the plasmid DNA extracted from PCR-positive clones was verified by Sanger sequencing to confirm that the sequence was in frame and had no mutations.

### 2.4. Expression and Purification of Recombinant Proteins

The expression of N_Δ121_ and full-length N proteins were conducted using the *E. coli* protein expression system. Briefly, a large-scale culture of *E. coli* (1 L) was performed in a shaker incubator (37 °C, 130 rpm) in LB broth containing 50 µg/mL ampicillin. When the optical density (OD) at 595 nm reached 1.0, expression of rN_Δ121_ and full-length N protein was induced with 0.1 M isopropyl-β-D-thiogalactopyranoside (IPTG). Three hours after induction, the *E. coli* culture fluid was centrifuged at 7000× *g* rpm at 4 °C for 30 min. The pellet was resuspended in denature-lysis-binding buffer containing 8 M urea, 20 mM sodium phosphate, and 30 mM imidazole and sonicated on ice for 5 min (5 s plateau, 5 s interval between pulses). The sonicated mixture was centrifuged at 14,000× *g* rpm at 4 °C for 30 min, and the supernatant was filtered with a 0.45 µm pore size filter before applying it to the histidine (His) tag affinity column. The expressed protein was double-purified using the His-tag affinity nickel (Ni^2+^) column (GE Healthcare Biosciences AB, Uppsala, Sweden) under 8 M urea in a denatured condition and His tag affinity cobalt (Co^2+^) column (GE Healthcare Biosciences AB, Uppsala, Sweden) without urea. Finally, the rN_Δ121_ and full-length N protein were eluted with 50 mM sodium phosphate, 300 mM sodium chloride, and 150 mM imidazole pH 7.4 elution buffer. The eluted true-peak fractions were pooled and stored at −30 °C before use. In total, 1 to 1.5 mg of purified protein was obtained per 1 L of *E. coli* culture, and its final concentration was 0.5 mg/mL.

### 2.5. Western Blot Analysis and Silver Staining

The expected sizes of SARS-CoV-2 rN_Δ121_ protein and full-length N protein were confirmed by Western blotting after SDS-PAGE. Immunostaining was performed using anti-His IgG (anti-6x His mouse monoclonal antibody [mAb] 4A12EU, Thermo Fisher Scientific, Waltham, MA, USA) and horseradish peroxidase (HRP)-conjugated goat anti-mouse IgG (American Qualex, CA, USA). The reaction was visualized by diaminobenzidine (DAB) substrate (Dojindo, Kumamoto, Japan). The purity was checked by silver staining (Cosmo Bio Co., Ltd., Tokyo, Japan) according to the manufacturer’s instructions.

### 2.6. In-House SARS-CoV-2 N Truncated and Full-Length N Proteins-Based IgG Indirect ELISAs

As described above, 177 plasma samples from the COVID-19 suspected cases and 155 pre-COVID-19 group serum samples were applied to evaluate our in-house IgG indirect ELISA. The optimal concentration of Co^2+^ purified SARS-CoV-2 rN_Δ121_ was determined by checkerboard titration, and 0.13 µg/µL showed low background. Hence, the Co^2+^ purified protein was diluted at 0.13 µg/100 µL/well [27] in carbonate buffer pH 9.6, then coated on a 96-well flat-bottom ELISA plate at 4 °C overnight. The same concentration was applied for SARS-CoV-2 full-length N protein-based IgG ELISA. Immulon 1B plate (Thermo Scientific, Rochester, NY, USA) was applied for these ELISAs to reduce the background color reaction. The following day, 100 µL/well of Block Ace (UK-1 B 80, Yukijirushi, Sapporo, Japan) was added to the plate, excluding the blank wells, and the plate was incubated at 37 °C for 1 h for blocking. After washing three times with PBS-Tween 20 (PBS-T), 100 µL/well of 1:200 diluted plasma/serum samples in Block Ace were applied in duplicate. The plate was incubated at 37 °C for 1 h. After washing three times with PBS-T, 100 µL/well of 1:10,000-diluted HRP-conjugated anti-human IgG (American Qualex, CA, USA) in PBS-T with a one-tenth volume of Block Ace was added. The plate was then incubated at 37 °C for 1 h and washed three times with PBS-T. Finally, 100 µL/well of *o*-phenylenediamine dihydrochloride (OPD) substrate (0.5 mg/mL) (Sigma Chemical, St. Louis, MO, USA) was added. The plate was incubated in the dark at room temperature for 30 min to 1 h before adding 100 µL/well of the stop solution (1N hydrochloric acid). The plate OD was read at 492 nm (Multiscan JX, Thermolab System, Tokyo, Japan). The mean OD of duplicate sample wells was calculated, including the SN (sample/negative control [NC]) ratio (mean sample OD ÷ mean NC OD). The negative control serum was selected among yellow fever vaccinated healthy volunteers collected in 2018. The endpoint IgG titers of the samples were also calculated using the standard curve of the high titer positive control serum. The standard curve was prepared using the 492 nm OD values of the positive control serum starting with a 200-fold dilution and followed by serial two-fold dilutions up to 1:2^11^ in PBS-T + 10% Block Ace.

### 2.7. Commercial SARS-CoV-2 S Protein-IgG Indirect ELISA

To evaluate the reliability of our in-house rN_Δ121_ IgG indirect ELISA, a commercial S protein-based IgG ELISA (Cell Signaling Technology, Danvers, MA, USA) was applied to the same samples following the manufacturer’s instructions. Briefly, the assay was a solid-phase ELISA that detects the binding of human IgG to full-length SARS-CoV-2 S protein. The mean absorbance at 450 nm of each duplicate sample was calculated. Sample values greater than 4.1 × NC absorbance, less than 3 × NC absorbance, and between 3 × NC and 4.1 × NC absorbance were considered positive, negative, and inconclusive, respectively.

### 2.8. Plaque Reduction Neutralization Test

A 50% plaque reduction neutralization test (PRNT_50_) was performed in biosafety level 3 laboratory (BSL-3) conditions. Briefly, human sera were heat-inactivated at 56 °C for 30 min and two-fold serially diluted (from 1:10 to 1:10,240) in 2% FCS MEM containing 0.2 mM nonessential amino acids. An equal volume of 100 PFU/200 µL/well virus (SARS-CoV-2 virus strain 2019-nCoV/Japan/TY/WK-521/2020) diluted in the same diluent as the serum sample was added with each diluted serum and incubated at 37 °C for 1 h in 5% CO_2_. Subsequently, 200 µL of the mixture was added in duplicate on confluent Vero E6 cells growing in a 24-well plate and incubated at 37 °C for 1 h in 5% CO_2_. Finally, 500 µL of 1.25% methylcellulose overlay medium prepared in 1% FCS with 2 times concentrated MEM containing 0.2 mM nonessential amino acids was added per well and incubated at 37 °C in 5% CO_2_. When CPE appeared on day 5, fixation with 4% paraformaldehyde solution was performed, and the plate was stained with 0.25% crystal violet. The reciprocal of the endpoint serum dilution that provided a 50% or greater reduction in the mean number of plaques relative to the control wells that contained no serum was defined as the PRNT_50_.

### 2.9. Statistical Analysis

Statistical analysis was performed using STATA software version 15.1. The cut-off value of the SN ratio was determined using the receiver operating characteristic (ROC) curve analysis at a 95% confidence interval (CI). The area under the ROC curve (AUC) and the sensitivity and specificity of the IgG indirect ELISA SN ratio were calculated based on the PRNT50 result. The SN ratio with the highest AUC was selected as the optimal cut-off point. Pearson’s chi-square test was used to compare the SN ratio to the PRNT50 titer using GraphPad Prism 9.1.1.

## 3. Results

### 3.1. Expression and Purification of the Recombinant SARS-CoV-2 Nucleocapsid Protein

**Figure 1:**
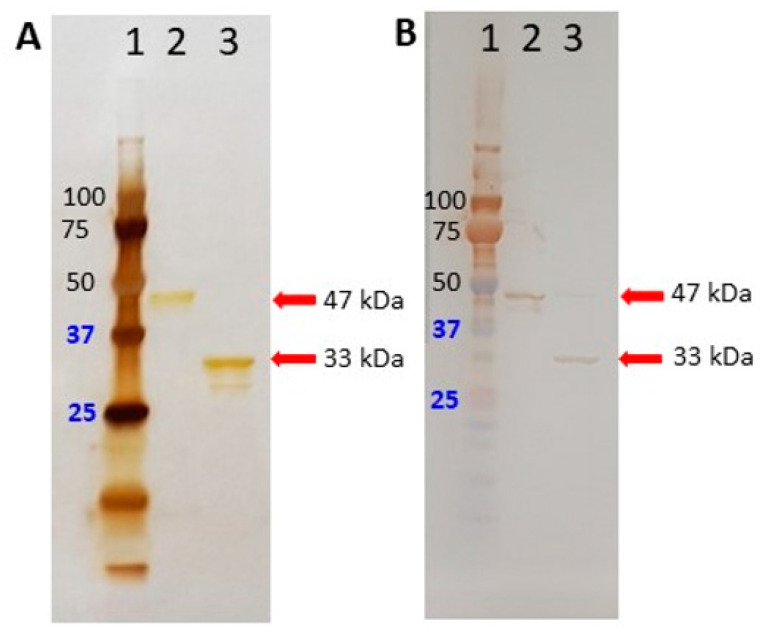
Expression and purification of SARS-CoV-2 rfull-length N and rN_Δ121_ proteins in Silver Staining (**A**), Western blotting (**B**). Lane 1, protein marker; lane 2, cobalt column purified rfull-length N protein; lane 3, cobalt column purified rN_Δ121_ protein in A and B. The purified size band of rN_Δ121_ and rfull-length N proteins is 33 kDa and 47 kDa, respectively.

In this study, we deleted the first 121 aa of SARS-CoV-2 N protein to reduce cross-reactivity with others HCoVs. Both truncated and full-length SARS-CoV-2 N proteins were successfully expressed in the *E. coli* protein expression system, purified by His-tag affinity chromatography column, and used as assay antigens to detect IgG against SARS-CoV-2. The reliability of SARS-CoV-2 N_Δ121_ protein-based IgG ELISA was compared with PRNT_50_, which is the gold standard method of coronaviruses serology [30,31]. Given that SARS-CoV-2 serological methods have focused on S protein [13,20], a commercial SARS-CoV-2 full-length S protein-based IgG indirect ELISA was used as the second gold standard. As a monomer and an oligomer, the SARS-CoV-2 N protein has a molecular mass of 51.38 kDa and >670 kDa, respectively [32,33]. However, the SDS-PAGE silver-stained gel (Figure 1A) and the Western blot (Figure 1B) revealed a 47 kDa and 33 kDa band sizes of full-length (lane 2) and truncated (lane 3) N proteins, respectively. This might be due to the 3D structure of N proteins affecting the faster migration. Additionally, additional bands were detected by anti-His below the 33 kDa band (Figure 1A, lane 3), which might be due to the degradation of rN protein [34].

### 3.2. SARS-CoV-2 N Truncated Protein-Based IgG ELISA

**Figure 2:**
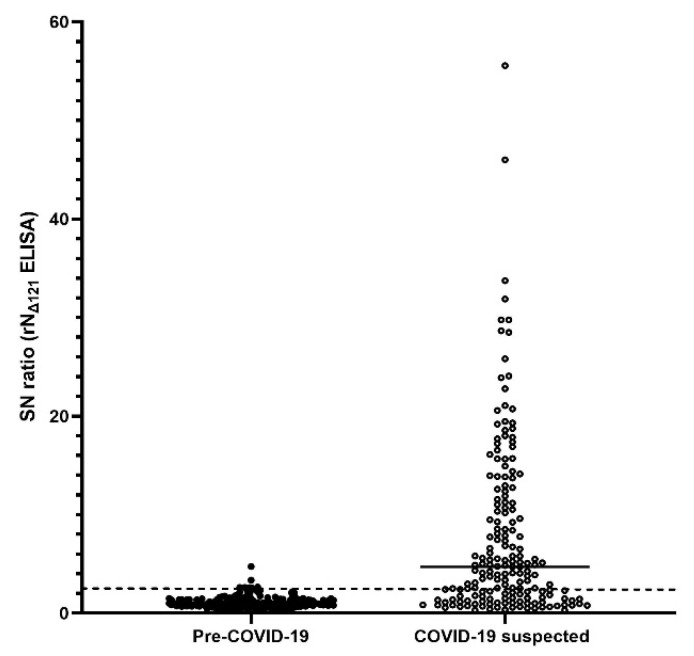
SARS-CoV-2 N_Δ121_ IgG ELISA. SN ratio distribution of pre-COVID-19 and COVID-19 suspected case samples. The broken line indicates the positive SN ratio cut-off. The continuous line indicates the SN ratio median value (4.7) of COVID-19 suspected case samples.

**Figure 3:**
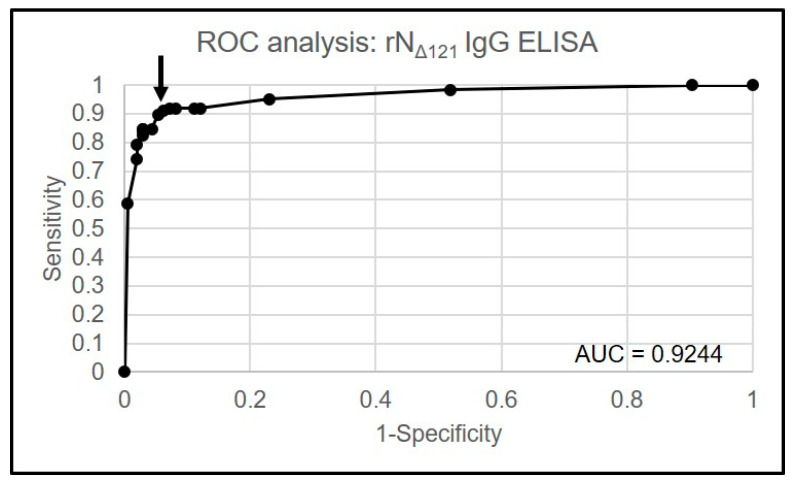
Receiver operating characteristic (ROC) curve of SARS-CoV-2 rN_Δ121_ IgG ELISA. The curve was generated by plotting the sensitivity and 1-specificity values of the SN ratio on the *y*-axis and *x*-axis, respectively. The arrow indicates the highest area under the ROC curve (AUC), which was 0.9244.

The purified rN_Δ121_ protein was used as an assay antigen for the anti-SARS-CoV-2 IgG indirect ELISA at the optimal concentration of 0.13 µg/100 µL/well. An SN ratio of 2.4 showed the highest AUC (0.9244), and was selected as the positive cut-off criterion (Figure 2 and Figure 3).

The positive cut-off IgG titer was considered 1:354, equivalent to 2.4 times the negative control IgG titer. Of the 177 COVID-19 suspected case samples, 118 were IgG positive and, 59 were negative, whereas 8 of 155 pre-COVID-19 samples were IgG positive and 147 were negative (Table 1).

### 3.3. SARS-CoV-2 SARS-CoV-2 Full-Length N Protein-Based IgG ELISA

To compare the relative performance between the SARS-CoV-2 N_Δ121_ and the full-length N protein-based IgG ELISA, the same concentration of the assay antigen (0.13 µG/100µL) and SN ratio cut-off criterion (2.4) were applied for SARS-CoV-2 full-length N protein-based IgG ELISA. With reference to the PRNT gold standard, 124 of 177 COVID-19 suspected case samples were IgG positive, while 17 of 155 pre-COVID-19 samples were positive and 138 were negative (Table 2).

Compared to the commercial S protein-based IgG ELISA, 124 of 177 and 16 of 155 were positive among COVID-19 suspected cases samples and pre-COVID-19 samples, respectively (Table 3).

### 3.4. SARS-CoV-2 S Protein-Based IgG ELISA

To compare the reactivity between the commercial SARS-CoV-2 S protein-based IgG indirect ELISA and our in-house rN_Δ121_ IgG indirect ELISA, all 332 samples were also assessed by S IgG ELISA. Among 177 COVID-19 suspected case samples, 121 were IgG positive, and 56 were IgG negative (Table 4). By contrast, only 2 of 155 pre-COVID-19 were IgG positive (Table 4), and 153 were IgG negative. No inconclusive results were found.

### 3.5. Plaque Reduction Neutralization Test

The PRNT_50_ titer ≥ 1:10 was applied as the positive cut-off criterion [30]. Of 177 COVID-19 suspected case samples, 124 samples were positive, with a PRNT_50_ titer ranging from 1:10 to 1:5120 (mode titer of positive samples, 1:160), and 53 samples were negative (<1:10 PRNT_50_ titer) (Table 5).

**Figure 4:**
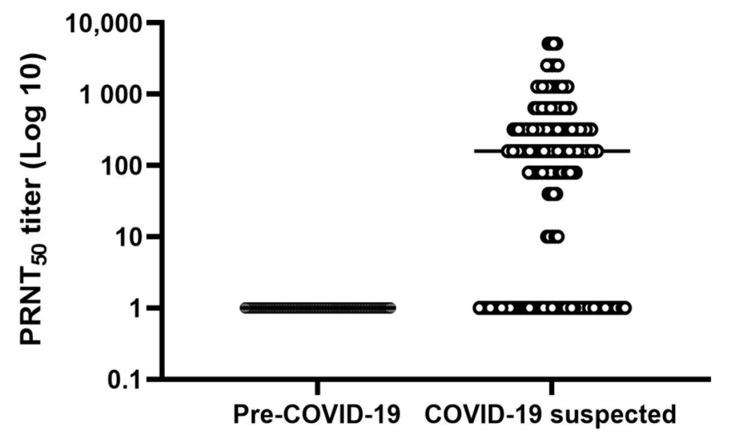
Distribution of PRNT_50_ titer against SARS-CoV-2. The open circle represents the PRNT_50_ titer distribution, and the black line indicates the mode titer (1:160) of positive COVID-19 suspected case samples. The filled circles show the PRNT_50_ titer distribution of pre-COVID-19 samples (<1:10).

As expected, the PRNT_50_ titers of all 155 pre-COVID-19 samples were below 1:10 (Figure 4 and Table 5).

### 3.6. Comparison of SARS-CoV-2 N Truncated Protein-Based IgG ELISA with Plaque Reduction Neutralization Test

**Figure 5:**
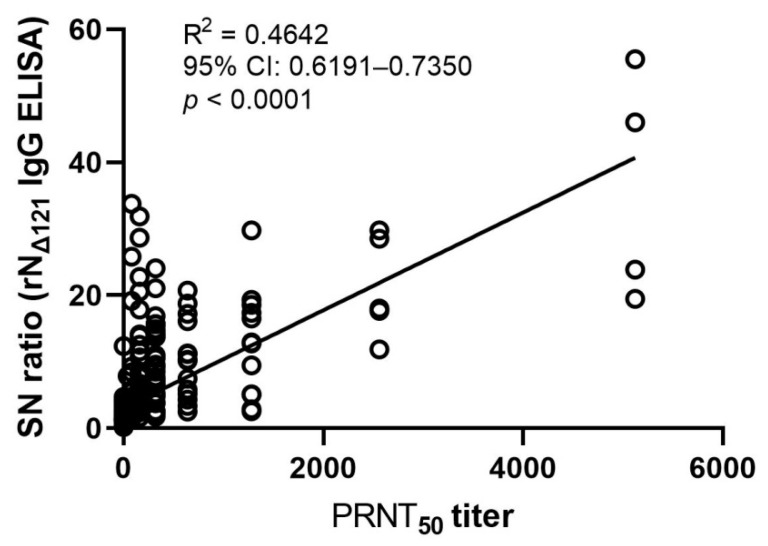
Correlation between in-house SARS-CoV-2 rN_Δ121_ IgG indirect ELISA SN ratio and PRNT_50_ titer. Pearson correlation (GraphPad Prism 9.1.1) was used to evaluate whether the SN ratio was correlated with the PRNT_50_ titer with a 95% confidence interval.

The performance of our in-house N_Δ121_ IgG ELISA was evaluated with the PRNT gold standard. Of 118 rN_Δ121_ IgG ELISA positive samples, 113 were confirmed positive by PRNT_50_, and 48 of 59 negative rN_Δ121_ IgG ELISA samples were confirmed negative by PRNT_50_ (Table 1). Compared with PRNT_50_ results, our in-house N_Δ121_ protein-based IgG ELISA showed 91.1% sensitivity, 93.8% specificity, 89.7% positive predictive value (PPV), 94.7% negative predictive value (NPV), and 92.8% concordance (Table 1). The correlation between the SN ratio of rN_Δ121_ IgG indirect ELISA and PRNT_50_ titer was statistically significant (R^2^ = 0.4642, 95% CI: 0.6191–0.7350, *p* < 0.0001) (Figure 5).

### 3.7. Comparison of SARS-CoV-2 N Truncated Protein-Based IgG ELISA with S IgG Protein-Based IgG ELISA

Among 118 rN_Δ121_ IgG ELISA positive COVID-19 suspected case samples, 113 were confirmed positive by S IgG ELISA, and 51 of 59 rN_Δ121_ IgG ELISA negative samples were confirmed negative by S IgG ELISA (Table 6).

Of the 155 pre-COVID-19 samples, 153 samples were confirmed negative by S IgG ELISA (Table 6). Compared with the commercial S protein-based IgG ELISA, our in-house N_Δ121_ protein-based IgG ELISA showed 91.9% sensitivity, 93.8% specificity, 89.7% PPV, 95.2% NPV, and 93.1% concordance (Table 6).

### 3.8. Comparison of SARS-CoV-2 S Protein-Based IgG ELISA with Plaque Reduction Neutralization Test

As for the rN_Δ121_ IgG ELISA, we evaluated the performance of the commercial S IgG ELISA with PRNT_50_ as the gold standard. Among 121 COVID-19 suspected IgG-positive samples, 119 were confirmed positive by PRNT, and 51 of 56 IgG-negative samples were confirmed negative by PRNT (Table 4). In comparison with the PRNT_50_ gold standard, S protein-based IgG ELISA showed 96.0% sensitivity, 98.1% specificity, 96.8% PPV, 97.6% NPV, and 97.3% concordance (Table 4).

## 4. Discussion

Recombinant nucleocapsid protein has been used for the serodiagnosis of several viruses, such as SARS-CoV [27,35], MERS-CoV [36], severe fever with thrombocytopenia syndrome virus (SFTSV) [37,38], Rift Valley fever virus [39], Nipah virus [40], and SARS-CoV-2 [41,42,43,44]. Although SARS-CoV-2 immunoassays and point-of-care assays are appearing in the market, comparative performance data are urgently needed for laboratory diagnosis and the public health response to COVID-19 [45].

In this study, we developed and evaluated an in-house SARS-CoV-2 rN_Δ121_ protein-based IgG ELISA. As indicated in the table below, our in-house SARS-CoV-2 N_Δ121_ protein-based IgG ELISA showed similar performance to the PRNT and S IgG ELISA (Table 7). Xiang et al. evaluated the performance of an in-house SARS-CoV-2 full N protein-based IgG indirect ELISA using 85 RT-PCR-confirmed COVID-19 samples and 60 negative control samples [46]. A similar study was conducted by Tehrani et al. with a larger sample size (100 RT-PCR confirmed COVID-19 cases vs. 300 pre-COVID-19 samples) [43]. Although the size of the assay antigen (truncated vs. full-length) and the gold standards are different (PRNT vs. RT-PCR), the sensitivity of our in-house rN_Δ121_ IgG indirect ELISA appeared much higher than that reported by Xiang et al. (Table 7). This could be due to the difference in ELISA sampling time, which was conducted earlier in the Xiang et al. study (13 days after RT-PCR) than in our study (1 month after RT-PCR/RT-LAMP). The differences in sensitivity, specificity, and concordance between Tehrani’s study and our results (Table 7) might be due to the disparity in the number of negative samples (300 vs. 155). Compared to SARS-CoV-2 full-length N protein, the rN_Δ121_ detected a low number of false-positive samples (13 vs. 21) (Table 1 and Table 2). Overall, the truncation improved the specificity of our in-house ELISA.

Previous reports demonstrated that antibody levels remain low or undetectable in mild or asymptomatic COVID-19 cases [47,48]. This study used plasma samples from mild, asymptomatic, and negative cases of COVID-19 collected one month after RT-PCR/RT-LAMP. Our results showed very few samples with high PRNT_50_ titers (Figure 4 and Table 3) or SN ratios of rN_Δ121_ IgG indirect ELISA (Figure 2), which could be explained by the weak correlation between the SN ratio and PRNT_50_ titer observed in our study (Figure 5).

To date, cross-reactivity with other human coronaviruses remains a significant concern of SARS-CoV-2 serological tests, including ELISA [13,49]. We deleted the first 121 aa of the N protein, which contain highly conserved N protein regions among coronaviruses [26,27]. Our in-house IgG rN_Δ121_ IgG ELISA detected a lower number of false-positive IgG sera than the full-length IgG ELISA (13 vs. 21) (Table 1 and Table 2). These false-positive IgG results could be due to pre-existing cross-reactive antibodies with common cold HCoVs [13], non-specific binding antibodies [50], or specific antibodies without neutralizing ability [51]. Our study has one limitation. We could not confirm whether false-positive IgG samples were cross-reactive antibodies to common cold HCoVs.

## 5. Conclusions

Population testing plays a key role in controlling the COVID-19 pandemic. We have developed a highly sensitive and specific SARS-CoV-2 N_Δ121_ protein-based IgG ELISA, which matches at 93% as concordances with two gold standard tests. Our in-house ELISA is cheaper, faster, and simpler than molecular tests. It can be scaled up and made into immunochromatographic kits, representing a valid option for routine diagnosis and surveillance of COVID-19.

## Figures and Tables

**Table 1:** Sensitivity and specificity of SARS-CoV-2 rNΔ121 IgG indirect ELISA with reference to PRNT_50_ ^*^.

COVID-19 Suspected			PRNT50	
		Positive	Negative	Total
rN_Δ121_ IgG ELISA	Positive	113	5	118
	Negative	11	48	59
	Total	124	53	177
Pre-COVID-19			PRNT50	
		Positive	Negative	Total
rN_Δ121_ IgG ELISA	Positive	0	8	8
	Negative	0	147	147
	Total	0	155	155

* Sensitivity, specificity, positive predictive value (PPV), negative predictive value (NPV), and concordance of 332 total samples were 91.1%, 93.8%, 89.7%, 94.7%, and 92.8%, respectively.

**Table 2:** Sensitivity and specificity of SARS-CoV-2 rN full-length IgG indirect ELISA with reference to PRNT_50_ ^*^.

COVID-19 Suspected			PRNT_50_	
		Positive	Negative	Total
rfull-length N IgG ELISA	Positive	120	4	124
	Negative	4	49	53
	Total	124	53	177
Pre-COVID-19			PRNT_50_	
		Positive	Negative	Total
rfull-length N IgG ELISA	Positive	0	17	17
	Negative	0	138	138
	Total	0	155	155

^*^ Sensitivity, specificity, PPV, NPV, and concordance of 332 total samples were 96.8%, 89.9%, 85.1%, 97.9%, and 92.8%, respectively.

**Table 3:** Sensitivity and specificity of SARS-CoV-2 rN full-length IgG indirect ELISA with reference to S IgG ELISA ^*^.

COVID-19 Suspected			S IgG ELISA	
		Positive	Negative	Total
rfull-length N IgG ELISA	Positive	120	4	124
	Negative	1	52	53
	Total	121	56	177
Pre-COVID-19			S IgG ELISA	
		Positive	Negative	Total
rfull-length N IgG ELISA	Positive	1	16	17
	Negative	0	138	138
	Total	1	154	155

* Sensitivity, specificity, positive predictive value (PPV), negative predictive value (NPV), and concordance of 332 total samples were 99.2%, 90.5%, 85.8%, 99.5%, and 93.7%, respectively.

**Table 4:** Sensitivity and specificity of SARS-CoV-2 S IgG indirect ELISA with reference to PRNT_50_^*^.

COVID-19 Suspected			PRNT_50_	
		Positive	Negative	Total
S IgG ELISA	Positive	119	2	121
	Negative	5	51	56
	Total	124	53	177
Pre-COVID-19			PRNT_50_	
		Positive	Negative	Total
S IgG ELISA	Positive	0	2	2
	Negative	0	153	153
	Total	0	155	155

^*^ Sensitivity, specificity, positive predictive value (PPV), negative predictive value (NPV), and concordance of 332 total samples were 96.0%, 98.1%, 96.8%, 97.6%, and 97.3%, respectively.

**Table 5:** PRNT titer distribution of COVID-19 suspected case samples and pre-COVID-19 samples.

	Pre-COVID-19	COVID-19 Suspected
PRNT_50_ titer	Number	Number
1:5120	0	4
1:2560	0	5
1:1280	0	12
1:640	0	14
1:320	0	29
1:160	0	33
1:80	0	18
1:40	0	4
1:20	0	0
1:10	0	5
<1:10	155	53
Total	155	177

**Table 6:** Sensitivity and specificity of SARS-CoV-2 rN_Δ121_ IgG indirect ELISA with reference to S IgG ELISA ^*^.

COVID-19 Suspected			S IgG ELISA	
		Positive	Negative	Total
rN_Δ121_ IgG ELISA	Positive	113	5	118
	Negative	8	51	59
	Total	121	56	177
Pre-COVID-19			S IgG ELISA	
		Positive	Negative	Total
rN_Δ121_ IgG ELISA	Positive	0	8	8
	Negative	2	145	147
	Total	2	153	155

^*^ Sensitivity, specificity, positive predictive value (PPV), negative predictive value (NPV), and concordance of 332 total samples were 91.9%, 93.8%, 89.7%, 95.2%, and 93.1%, respectively.

**Table 7:** Comparison between in-house full-length N, N_Δ121_ and other studies rSARS-CoV-2 IgG ELISAs.

In-House rSARS-CoV-2 ELISA	Sensitivity (%)	Specificity (%)	PPV (%)	NPV (%)	Concordance (%)	Reference Test
N_Δ121_	91.1	93.8	89.7	94.7	92.8	PRNT
N_Δ121_	91.9	93.8	89.7	95.2	93.1	Commercial S IgG ELISA
Full-length N	96.8	89.9	85.1	97.9	92.5	PRNT
Full-length N (Xiang et al.)	83.3	95.0	94.8	83.8	88.9	RT-PCR
Full-length N (Tehrani et al.)	89.0	98.0			96.0	RT-PCR

## Data Availability

The data presented in this study are available on request from the corresponding author.
